# Characteristics of the Supersonic Combustion Coherent Jet for Electric Arc Furnace Steelmaking

**DOI:** 10.3390/ma12213504

**Published:** 2019-10-25

**Authors:** Fei Zhao, Rong Zhu, Wenrui Wang

**Affiliations:** 1 Key Laboratory of Fluid Interaction with Material, University of Science and Technology Beijing, Beijing 100083, China; 2 School of Metallurgical and Ecological Engineering, University of Science and Technology Beijing, Beijing 100083, China; rongzhu1201@163.com; 3 School of Mechanical Engineering, University of Science and Technology Beijing, Beijing 100083, China; gmbitwrw@ustb.edu.cn

**Keywords:** coherent jet, supersonic shrouding gas, supersonic combustion, flow-field characteristics, EAF steelmaking

## Abstract

Herein, a supersonic combustion coherent jet is proposed based on current coherent jet technology to improve the impact capacity of a coherent jet and increase the stirring intensity of the electric arc furnace (EAF) bath. Further, numerical simulations and an experimental analysis are combined to study the supersonic combustion coherent jet characteristics, including the Mach number, dynamic pressure, static temperature, vorticity, and turbulence intensity, in the EAF steelmaking environment. The results show that the supersonic combustion coherent jet exhibits stable combustion in a high-temperature EAF steelmaking environment. The supersonic combustion flame generated by the supersonic shrouding fuel gas can envelop the main oxygen jet more effectively than current coherent jets. Furthermore, the velocity attenuation, vorticity, and turbulence intensity performances of the supersonic combustion coherent jet are better when compared with those of the current coherent jet. The velocity core length of the main oxygen jet for the supersonic combustion coherent jet is 30% longer than that of the current coherent jet, resulting in an improved impact capacity and stirring intensity of the molten bath.

## 1. Introduction

The steelmaking process requires continuous stirring to promote the oxidation of elements in molten steel, such as carbon, silicon, phosphorus, and manganese [1,2]. The stirring intensity of a supersonic oxygen jet supplied to a molten bath is an important factor in electric arc furnace (EAF) steelmaking [3,4]. The potential to further improve the stirring intensity and impact capacity of a supersonic oxygen jet has gained the attention of many researchers. Common supersonic oxygen jet technology has been eliminated because of its rapid velocity attenuation and weak impact capacity; currently, the coherent jet plays an important role during the EAF steelmaking process [5,6,7,8]. Here, the high-temperature flame of the coherent jet envelops the main oxygen jet, reducing the entrainment of the supersonic main oxygen jet to external ambient gas, delaying the velocity attenuation of the main oxygen jet, and enhancing the impact capacity to the molten bath. The coherent jet characteristics are influenced by the velocity, composition, and temperature of the shrouding fuel gas, and have been investigated by many researchers [9,10,11]. Tang et al. [12,13] investigated the effect of fuel input on the coherent jet length for three different fuel types (i.e., blast furnace gas, natural gas, and coke oven gas), and the results indicated that low molecular weight or gas density of the shrouding fuel would increase the potential core length of the main oxygen jet. Further, Odenthal et al. [14] proposed a coherent jet nozzle with a combustor structure that allowed the shrouding gas to burn in the combustor, with the high-temperature and high-speed combustion products enveloping the supersonic main oxygen jet and improving the impact capacity to the molten pool. Sumi et al. [15,16] and Klioutchnikov et al. [17] analyzed coherent jet characteristics under high-temperature and low-pressure conditions via a numerical simulation and an experimental method. The results showed that the velocity attenuation of the main oxygen jet was restrained and that the potential core length was extended in a high-temperature and low-pressure environment. Alam et al. [18,19,20] studied the influence of the shrouding gas parameters on the main oxygen jet velocity distribution and depth of penetration. The depth of penetration and liquid free surface velocity were observed to increase with an increase in the shrouding gas flow rate. These aforementioned studies primarily focused on coherent jets with a subsonic shrouding gas; however, some studies have focused on coherent jets with a supersonic shrouding gas (either high-temperature air or oxygen). Liu et al. [21,22] simulated the flow fields of the coherent jet with a shrouding Laval nozzle structure and studied the effects of the shrouding Mach number and ambient temperature on the coherent jet characteristics with a supersonic shrouding gas. Here, the supersonic combustion coherent jet with a supersonic shrouding fuel gas is proposed to improve the impact capability based on the aforementioned research results. Furthermore, the supersonic combustion coherent jet characteristics are studied in detail by combining the numerical simulation and experimental method because only some studies have investigated coherent jets with a supersonic shrouding fuel gas.

## 2. Experimental Equipment and Numerical Simulations

### 2.1. Experimental Equipment

**Figure 1:**
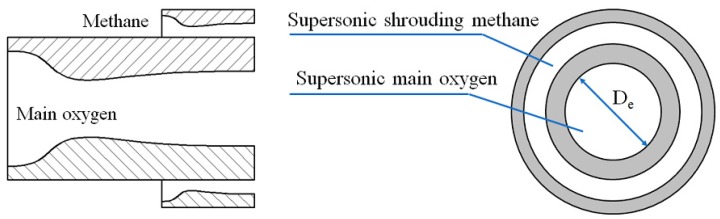
Structure of the supersonic combustion coherent jet nozzle.

**Figure 2:**
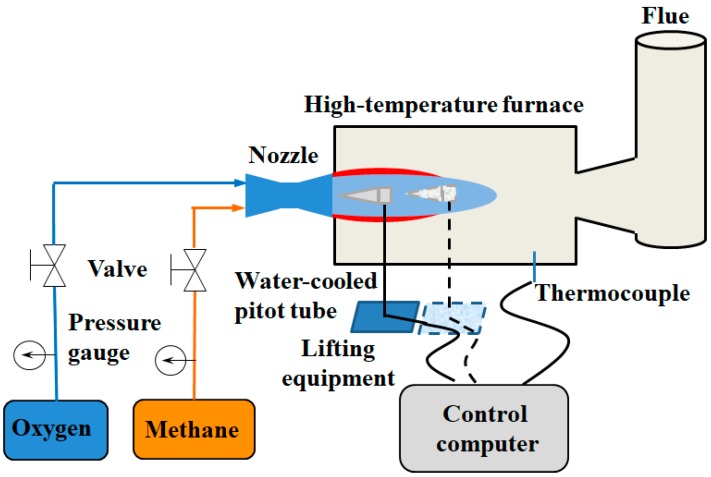
Schematic of the experimental apparatus.

The structure of the supersonic combustion coherent jet nozzle employed in the experiment is shown in Figure 1. The main oxygen and shrouding gas nozzles are Laval nozzles. The shrouding gas nozzle exhibits an annular design and surrounds the supersonic main oxygen nozzle, and both the Laval nozzles are designed with an exit Mach number of 2.0. The exit diameter of the supersonic oxygen jet is 30.66 mm, expressed as D_e_ in Figure 1. Figure 2 schematically depicts the experimental apparatus. Oxygen and methane are the gas sources, with methane serving as the supersonic shrouding gas. Further, the pressure and temperature of the supersonic main oxygen jet are measured using a water-cooled pitot tube and thermocouple, respectively, which are fixed to the lifting equipment. Subsequently, the pressure and temperature at different positions on the axis of the main oxygen jet are measured by changing the position of the lift equipment, and the temperature of the high-temperature furnace is measured using a built-in thermocouple. The Mach number of the main oxygen jet is calculated using Equations (1) and (2) [23].

(1) $$Ma=\sqrt{\frac{2}{\kappa -1}\left[{\left(\frac{P_{0}}{P_{s}}\right)}^{\left(\kappa -1\right)/\kappa }-1\right]}$$

and

(2) $$\frac{T_{s}}{T_{0}}=\frac{1}{1+r\frac{\kappa -1}{2}Ma^{2}}$$

where *Ma* is the Mach number of the supersonic main oxygen jet; *P_0_* and *P_s_* are the total and static pressures of the main oxygen jet, respectively (in Pa); *κ* is the heat capacity ratio; *T_0_* and *T_s_* are the total and static temperatures of the main oxygen jet, respectively (in K); and *r* is the temperature recovery coefficient.

### 2.2. Numerical Simulation

The Reynolds-averaged Navier–Stokes equations [24] are used for the computational fluid dynamics simulations. Further, the averaged mass, momentum, and energy equations can be expressed as follows:

(3) $$\frac{\partial \rho }{\partial t}+\frac{\partial }{\partial x_{i}}\left(\rho u_{i}\right)=0,$$

(4) $$\frac{\partial }{\partial t}\left(\rho u_{i}\right)+\frac{\partial }{\partial x_{j}}\left(\rho u_{i}u_{j}\right)=-\frac{\partial p}{\partial x_{j}}+\frac{\partial }{\partial x_{j}}\left[\mu \left(\frac{\partial u_{i}}{\partial x_{j}}+\frac{\partial u_{j}}{\partial x_{i}}-\frac{2}{3}\delta_{ij}\frac{\partial u_{k}}{\partial x_{k}}\right)\right]+\frac{\partial }{\partial x_{j}}\left(-\rho \bar{u_{i}^{\prime }u_{j}^{\prime }}\right),$$

(5) $$\frac{\partial }{\partial t}\left(\rho E\right)+\frac{\partial }{\partial x_{i}}\left[u_{i}\left(\rho E+p\right)\right]=\frac{\partial }{\partial x_{j}}\left(k_{eff}\frac{\partial T}{\partial x_{j}}+u_{i}{\left(\tau_{ij}\right)}_{eff}\right)+S_{h},$$

and

(6) $$\frac{\partial }{\partial t}\left(\rho E\right)+\frac{\partial }{\partial x_{i}}\left[u_{i}\left(\rho E+p\right)\right]=\frac{\partial }{\partial x_{j}}\left(k_{eff}\frac{\partial T}{\partial x_{j}}+u_{i}{\left(\tau_{ij}\right)}_{eff}\right)+S_{h}$$

where *ρ* is the fluid density (in kg·m^−3^); *u**_i_***, *u**_j_***, and *u**_k_*** are the velocity components in the *i*, *j*, *k* directions, respectively (in m·s^−1^); *P* is the fluid pressure (in Pa); *μ* and *μ_t_* are the molecular and turbulence viscosities, respectively (in Pa·s); *k* is the turbulent kinetic energy (in m^2^·s^−2^); *E* is the total energy (in J); *k_eff_* is the effective thermal conductivity (in W·m^−1^·K^−1^); *T* is the fluid temperature (in K); *τ_ij_* is the viscous stress (in N·s^−2^); and *S_h_* is the internal energy source.

Previous research has shown that the *k*-*ω* shear-stress transport model is appropriate for supersonic jet numerical simulations [25], which was originally developed by Menter [26] to effectively blend the robust and accurate formulation of the *k*-*ω* model in the near-wall region with the free-stream independence of the *k*-*ε* model in the far field:

(7) $$\frac{\partial }{\partial t}\left(\rho k\right)+\frac{\partial }{\partial x_{i}}\left(\rho ku_{i}\right)=\frac{\partial }{\partial x_{j}}\left[\Gamma_{k}\frac{\partial k}{\partial x_{j}}\right]+G_{k}-Y_{k}+S_{k}$$

and

(8) $$\frac{\partial }{\partial t}\left(\rho \omega \right)+\frac{\partial }{\partial x_{i}}\left(\rho \omega u_{i}\right)=\frac{\partial }{\partial x_{j}}\left[\Gamma_{\omega }\frac{\partial \omega }{\partial x_{j}}\right]+G_{\omega }-Y_{\omega }+D_{\omega }+S_{\omega }$$

where *G_k_* is the turbulent kinetic energy generated by the mean velocity gradient (in J); *G_ω_* is the turbulent kinetic energy generated by *ω* (in J); *Y_k_* and *Y_ω_* are the *k* and *ω* dissipation due to the turbulence, respectively (in J); *Γ_k_* and *Γ_ω_* represent the effective diffusivities of *k* and *ω*, respectively; *D_ω_* is the damping cross-diffusion phase; and *S_k_* and *S_ω_* are custom source phases.

### 2.3. Simulation Details

**Figure 3:**
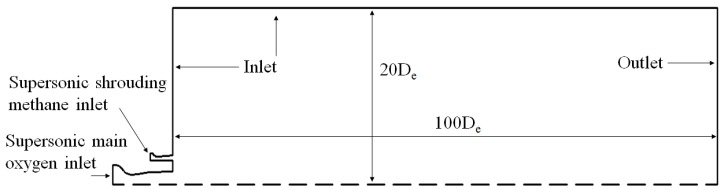
Computational domain with boundary conditions.

The mathematical model of the supersonic combustion coherent jet is shown in Figure 3. The computational domain includes the main oxygen Laval nozzle, shrouding gas Laval nozzle, and jet-spreading region. Further, the length and width of the jet-spreading region are 100 and 20 times the nozzle exit diameter, respectively. The axisymmetric swirl model is selected to perform the calculation. The second-order upwind scheme is used to discretize the equations and improve the simulation accuracy. The species transport model and Chemkin combustion mechanism are used to accurately estimate the temperature field. The residuals are set to <10^6^ for energy and 10^5^ for all the remaining variables. Table 1 presents the boundary conditions used in simulations. Three different jets (i.e., the supersonic, coherent, and supersonic combustion coherent jets) are simulated. Hereafter, the supersonic combustion coherent jet is termed as SC coherent jet for achieving simplicity.

### 2.4. Grid Independence Test

**Figure 4:**
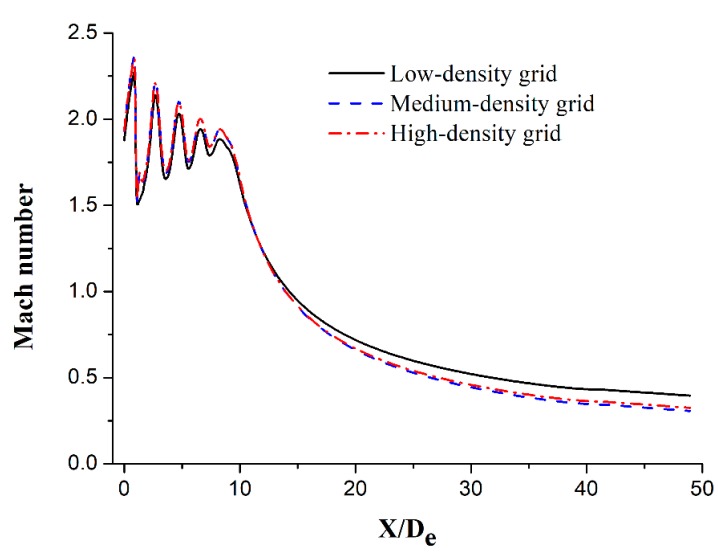
Axial Mach number of the main oxygen jet for different grid levels.

An important factor that affects the simulation results is the grid quality. The accuracy of the simulation results is proportional to the grid quality. Further, a grid independence test was conducted under different grid conditions to eliminate the effect of the grid quality on the simulation results. The axial Mach number distribution results for the low- (104,900 cells), medium- (198,000 cells), and high-density (323,000 cells) grids are shown in Figure 4, which exhibits similar Mach number distribution trends, and the axial Mach number distribution for the medium-density grid is observed to be almost identical to that for the high-density grid. However, some differences can be observed between the medium- and low-density grid results. Therefore, the medium-density grid was selected as the computational grid in this study to reduce the required computational time.

## 3. Results and Discussion

### 3.1. Mach Number Distribution

**Figure 5:**
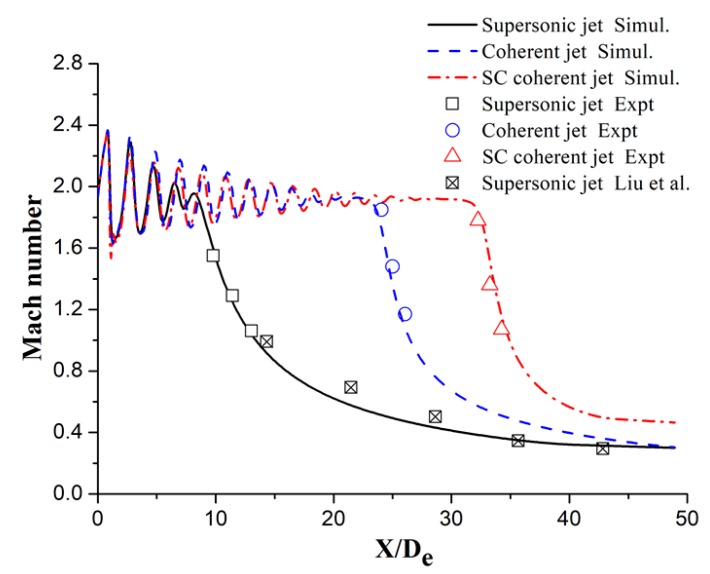
Axial Mach number of the main oxygen jet for different jets.

Figure 5 shows the Mach number distributions of the main oxygen jet for the three different jets. *D_e_* is the nozzle exit diameter of the main oxygen jet and the abscissa where *X*/*D_e_* = 0 is considered to be the nozzle exit plane. Further, the Mach number distribution trends of the main oxygen jet are observed to be similar for the three jets. The Mach numbers of the main oxygen jet for the three jets exhibit repeated fluctuations after exiting the nozzle, and the Mach number fluctuations are based on the designed Mach number of the nozzle exit. The fluctuating Mach number distribution decreases with the forward movement of the main oxygen jet. The velocity core length of the main oxygen jet for the supersonic jet is short (~9*D_e_*), and the Mach number of the main oxygen jet rapidly decreases after the velocity core length is reached. The velocity core length of the main oxygen jet for the coherent jet is longer than that for the supersonic jet, reaching 25*D_e_*, because of the shrouding gas flame protection. The velocity core length of the main oxygen jet for the SC coherent jet increases by ~30% when compared with that of the coherent jet, reaching 34*D_e_*. The supersonic combustion flame produced by the supersonic shrouding methane gas more effectively envelops the main oxygen jet and extends the velocity core length of the main oxygen jet more when compared with the subsonic shrouding gas flame. The numerical simulation results are in good agreement with the experimental results and the literature value [27]. The differences between the numerical simulation and experimental results do not exceed 8%. However, the difference between the numerical simulation and the literature value is slightly larger because the setup of the turbulence model, the method of meshing, and the selection of a discrete method will slightly affect the calculation results. Jones and Whitelaw [28] denoted the discrepancies in velocity and temperature contours during the simulation calculation process; however, these discrepancies will not affect the analysis of the final results.

**Figure 6:**
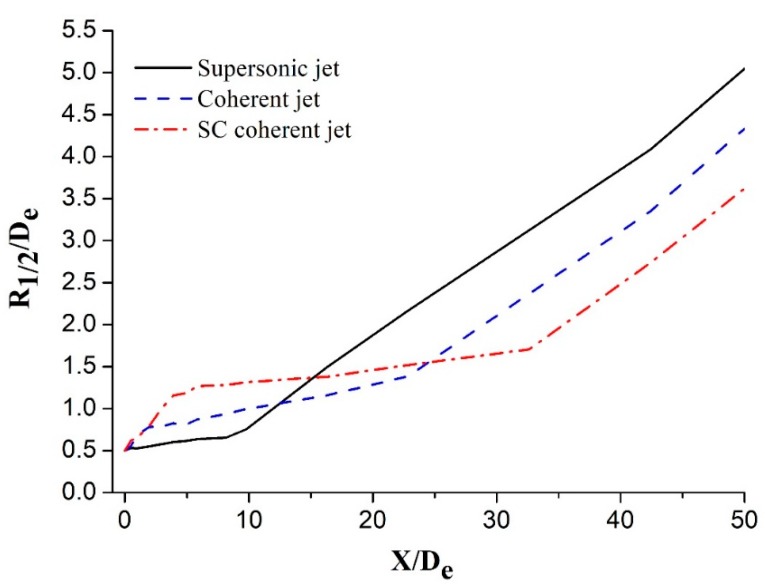
Half-jet width of the main oxygen jet for different jets.

Figure 6 shows the half-jet width (*R*_1/2_) distribution of the main oxygen jet at different axis positions; further, *R*_1/2_ denotes the radial distance where the jet velocity is half the axial velocity. *R*_1/2_/*D_e_* is the ratio of the half-jet width to the main oxygen nozzle exit diameter. The *R*_1/2_ distribution of the supersonic jet is different when compared with those of the coherent and SC coherent jets. The *R*_1/2_ distribution of the supersonic jet can be divided into the following two stages: an initial gradual increase in *R*_1/2_ after leaving the nozzle and then a rapid increase at a high rate. Further, the *R*_1/2_ distributions of the coherent and SC coherent jets can be classified into three stages. The *R*_1/2_ distribution of the coherent jet initially rapidly increases to *X/D_e_* = 2, then slowly increases to *X/D_e_* = 22, and finally increases at a certain high rate. The *R*_1/2_ distribution of the SC coherent jet is slightly different from that of the coherent jet in the first two stages. The *R*_1/2_ distribution of the SC coherent jet rapidly increases over a longer distance during the first stage, reaching a higher value compared to the coherent jet results. Subsequently, the *R*_1/2_ distribution of the SC coherent jet gradually increases at a lower rate and over a longer distance than the coherent jet because the main oxygen jet is more effectively enveloped by the supersonic combustion flame. The entrainment of the supersonic main oxygen jet in the external gas is reduced, and the radial expansion of the jet velocity is inhibited. The *R*_1/2_ distribution of the SC coherent jet is lower than that of the coherent jet during the final stage.

### 3.2. Pressure Distribution

**Figure 7:**
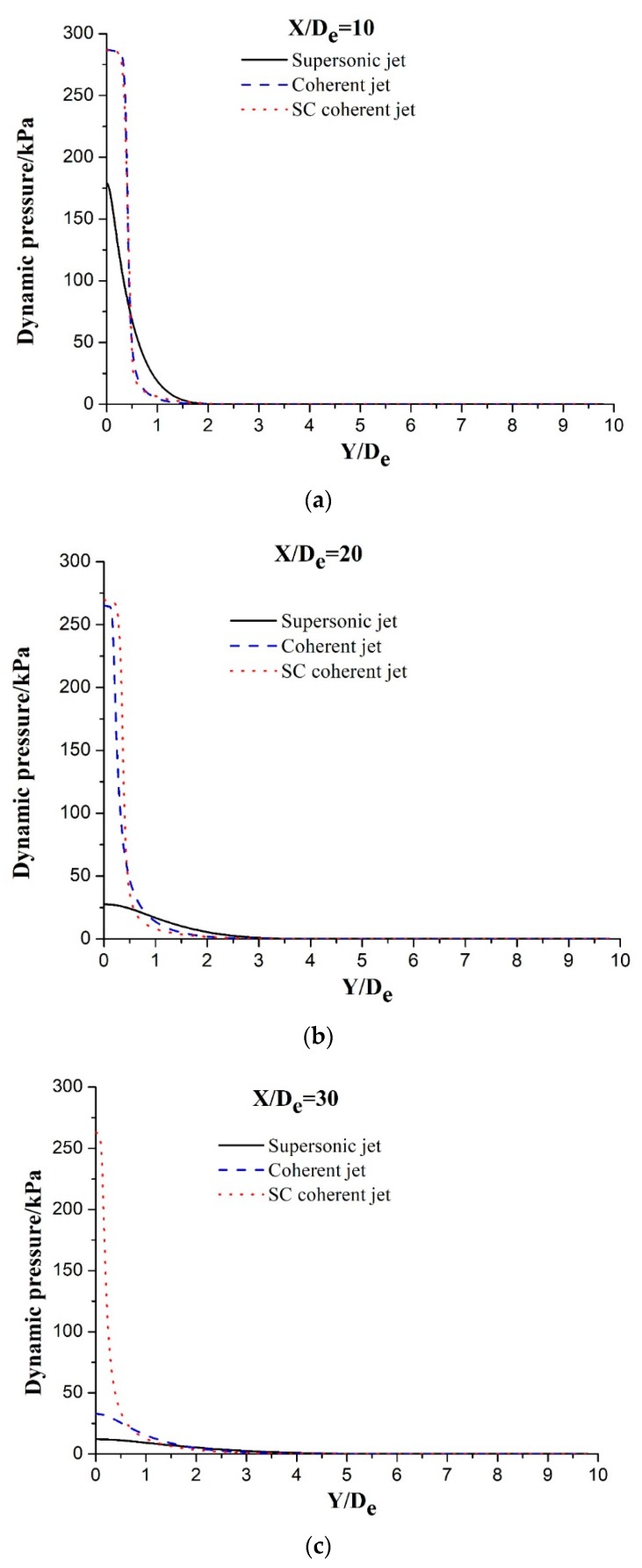
The radial dynamic pressure distribution at different axial locations in the case of different jets: (**a**) supersonic jet; (**b**) coherent jet; and (**c**) SC coherent jet.

The velocity decreases to 0 m·s^−1^ when the supersonic jet is completely blocked, and its kinetic energy is converted into dynamic pressure energy. The impact capacity to the molten bath is determined based on the dynamic pressure of the supersonic jet. Figure 7 depicts the radial dynamic pressure distribution at different axial locations for three different jets. All the dynamic pressure distribution trends of the main oxygen jet decrease along the radial direction when *X*/*D_e_* = 10, as shown in Figure 7a. The maximum dynamic pressures of the main oxygen jet are 179.0, 286.7, and 287.1 kPa for the supersonic, coherent, and SC coherent jets, respectively, and almost identical dynamic pressures can be observed in the cases of the coherent and SC coherent jets. The jet dynamic pressure continuously decreases with the movement of the main oxygen jet position. Further, the dynamic pressures for the three aforementioned jets are 27.6, 265.3, and 269.6 kPa, respectively, when *X*/*D_e_* = 20, which equate to decreases in dynamic pressure of 84.6%, 7.5%, and 6.1%, respectively. The dynamic pressure attenuation for the SC coherent jet is the lowest among the three jets. The decreases in dynamic pressure for the three jets from *X*/*D_e_* = 10 to 30 are 93.3%, 88.5%, and 8.4%, respectively. These results indicate that the dynamic pressure of the main oxygen jet for the SC coherent jet is the largest, whereas its dynamic pressure attenuation is the slowest. The supersonic combustion flame effectively envelops the main oxygen jet, reduces the entrainment of the main oxygen jet into the external gas, delays the velocity attenuation of the main oxygen jet, and causes the main oxygen jet to exhibit a high dynamic pressure.

### 3.3. Temperature Distribution

**Figure 8:**
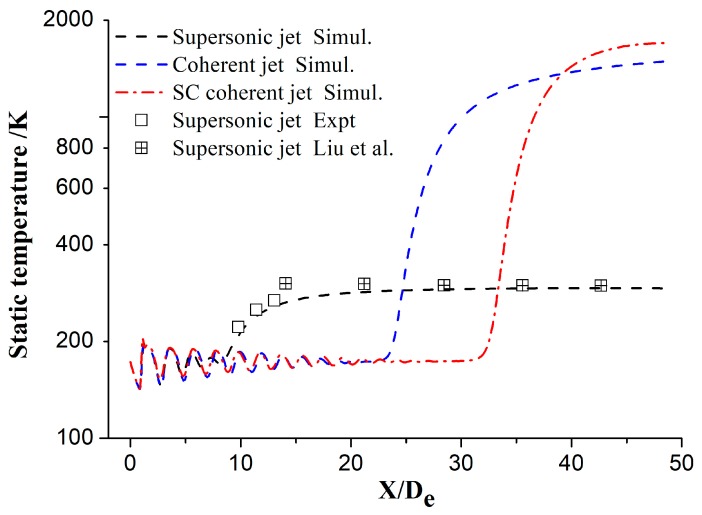
Static temperature of the main oxygen jet for different jets.

Figure 8 shows the axial static temperature distribution for different jets. The static temperature of the main oxygen jet initially fluctuates and subsequently trends to the ambient temperature. The axial static temperature for the supersonic jet remains unchanged below *X/D_e_* = 9, denoting that the temperature core length of the main oxygen jet for the supersonic jet is 9*D_e_*. However, the temperature core lengths for the coherent and SC coherent jets are 22*D_e_* and 32*D_e_*, respectively. The flame generated by the shrouding gas can effectively protect the main oxygen jet and avoid heat exchanges with the external ambient gas, maintaining low temperature over a long distance. Further, the temperature core length for the SC coherent jet is significantly longer than that for the coherent jet. These observations indicate that the supersonic combustion flame can more effectively envelop the main oxygen jet than the subsonic flame. Similar to the velocity distribution of the main oxygen jet, the simulated static temperature values are in good agreement with the experimental and literature values [27]; however, the difference between the simulated and literature values is slightly larger than the difference between the simulated and experimental values.

**Figure 9:**
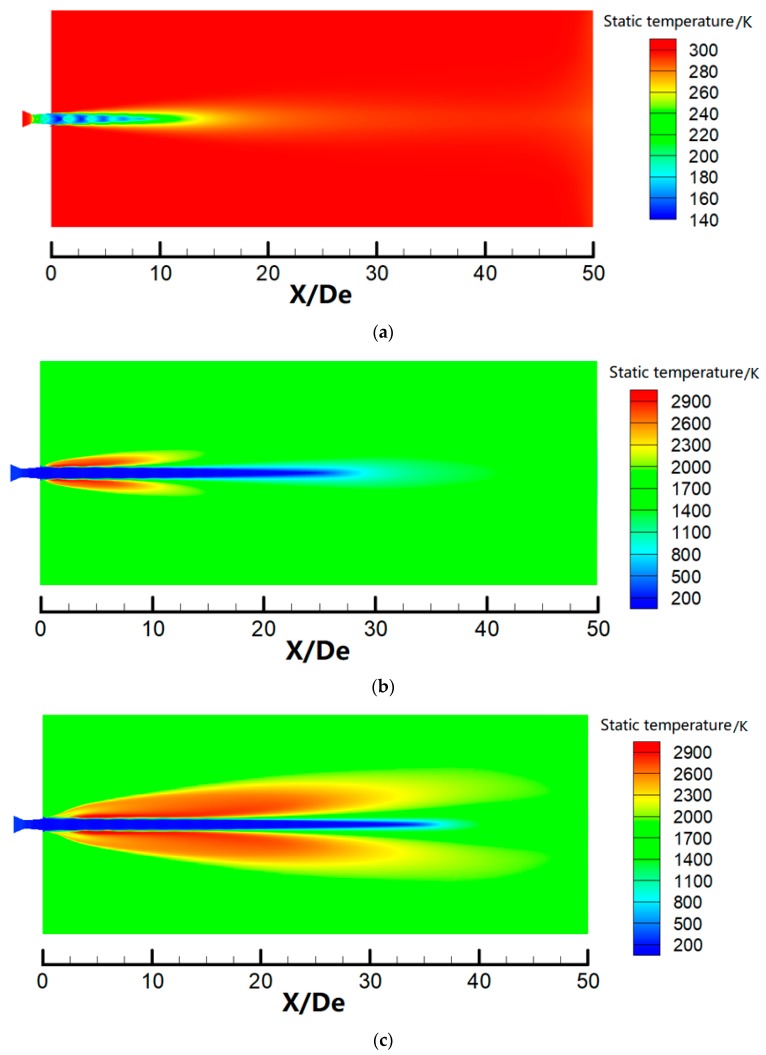
Static temperature contours for three jets: (**a**) supersonic jet; (**b**) coherent jet; and (**c**) SC coherent jet.

Figure 9 denotes the static temperature contours for three different jets. The main oxygen jet for the supersonic jet easily exchanges heat with the external ambient gas under an ambient temperature, and the temperature of the main oxygen jet quickly reaches the ambient temperature. When the shrouding gas flame surrounds the main oxygen jet, the shrouding gas flame isolates the main oxygen jet from the external environment, such that the main oxygen jet exchanges heat with the shrouding gas instead of the external ambient gas. The heat exchange between the main oxygen jet and the shrouding gas flame is weaker than that with the external still gas because the shrouding gas flame is emitted at a certain speed. The shrouding gas flame can increase the temperature core length of the main oxygen jet. The faster the velocity of the shrouding gas, the less heat exchanges with the main oxygen jet, with the smallest interaction being observed between the supersonic combustion flame and main oxygen jet for the SC coherent jet. Papamoschou and Roshko [29] indicated that the mixed layer thickness between the main oxygen jet and the external gas decreased with decreasing gas density in the external environment. When compared with Figure 9b,c, the SC coherent jet forms a lengthier and wider high-temperature flame surrounding the main oxygen jet. Further, the gas density around the main oxygen jet is reduced, and the mixing effect between the main oxygen jet and the external gas is weakened. Therefore, the SC coherent jet exhibits the longest velocity and temperature core length of the main oxygen jet.

### 3.4. Vorticity and Turbulence Intensity

**Figure 10:**
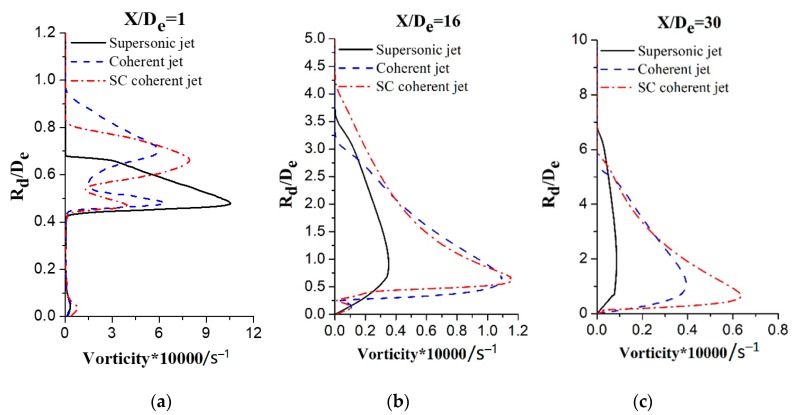
Vorticity magnitude radial distributions at different axial locations for three jets: (**a**) supersonic jet; (**b**) coherent jet; and (**c**) SC coherent jet.

Figure 10 shows the vorticity magnitude in the radial direction when *X/D_e_* = 1, 16, and 30 for different jets, where *R_d_/D_e_* is the ratio of the radial distance to the main oxygen nozzle exit diameter. The vorticity magnitude for the supersonic jet is considerably different when compared with magnitudes for the coherent and SC coherent jets after leaving the Laval nozzle, as depicted in Figure 10a. Only one peak vorticity magnitude can be observed for the supersonic jet as the main oxygen jet leaves the nozzle exit and mixes with still air at the periphery, and rotational flow can be observed at the periphery of the jet because of the large velocity gradient in that region. Two vorticity magnitude peaks can be observed for the coherent and SC coherent jets of which the first peak can be attributed to the mixing of the supersonic main oxygen jet with the supersonic shrouding gas and the second peak can be attributed to the mixing of the shrouding gas with the external ambient gas. However, the peak values are different for the coherent and SC coherent jets. While the Mach numbers of the supersonic shrouding gas and main oxygen jet are the same for the SC coherent jet, the Mach number of the shrouding gas for the coherent jet is only 0.8, considerably lower than that of the main oxygen jet. Therefore, the entrainment of the main oxygen jet into the supersonic shrouding gas is weaker than the entrainment into the subsonic shrouding gas. Hence, the first peak of the vorticity magnitude for the SC coherent jet is lower than that for the coherent jet and the second peak for the SC coherent jet is higher than that for the coherent jet because the supersonic shrouding gas exhibits strong kinetic energy and mixing ability because of its high velocity. The distribution trends of the vorticity magnitude for the three jets are observed to be similar when *X/D_e_* = 16 as the main oxygen jet continues to move forward. The velocity attenuation of the main oxygen jet for the supersonic coherent jet is the slowest, whereas the impact capacity to the external ambient gas is the strongest. Therefore, the vorticity magnitude of the main oxygen jet for the SC coherent jet is the largest, followed by the coherent jet, and the supersonic jet exhibits the smallest vorticity magnitude. The vorticity of the main oxygen jet for the coherent jet is considerably lower than that for the SC coherent jet when *X*/*D_e_* = 30, as shown in Figure 10c. 

**Figure 11:**
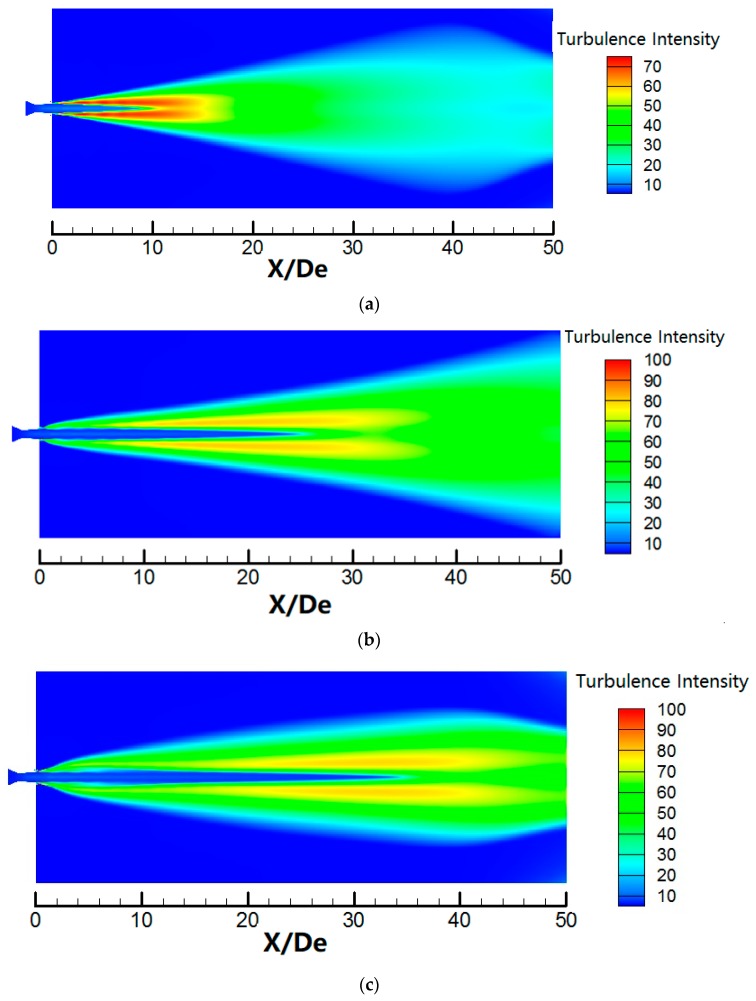
Turbulence intensity contours for three jets: (**a**) supersonic jet; (**b**) coherent jet; and (**c**) SC coherent jet.

Figure 11 shows the turbulence intensity contours for different jets. The turbulence intensity of the main oxygen jet reaches 70 in the external boundary of the supersonic main oxygen jet because of the mixing of the main oxygen jet with the external ambient gas, as shown in Figure 11a. The length of the low-turbulence intensity of the main oxygen jet is approximately *X/D_e_* = 9. The main oxygen jet initially mixes with the shrouding gas and subsequently mixes with the external ambient gas in the case of the coherent jet, as shown in Figure 11b. The main oxygen jet is enveloped by the shrouding gas flame and maintains low-turbulence intensity over a long distance, with the length of the low-turbulence intensity area reaching 25*D_e_*, because the shrouding gas effectively delays the mixture of the main oxygen jet and external ambient gas. The low-turbulence intensity length of the main oxygen jet for the SC coherent jet considerably exceeds that for the coherent jet, reaching *X/D_e_* = 35, indicating that the supersonic combustion flame can more effectively envelop the main oxygen jet and avoid the mixing of the main oxygen jet and external ambient gas, delaying the increase in the turbulence intensity of the main oxygen jet.

## 4. Conclusions

The flow-field characteristics of the supersonic, coherent, and supersonic combustion coherent jets are studied and analyzed via numerical simulations and an experimental method to investigate the ability to improve the EAF steelmaking process. The numerical simulation results are in good agreement with the experimental and literature results, and the following conclusions are obtained.

Stable combustion of the SC coherent jet can be realized in a high-temperature EAF steelmaking environment. The supersonic combustion flame generated by the supersonic shrouding fuel gas envelops the main oxygen jet more effectively than the subsonic shrouding gas in the case of the coherent jet, resulting in improved velocity, temperature, vorticity, and turbulence velocity characteristics when the SC coherent jet is used. The velocity attenuation of the main oxygen jet in the case of the SC coherent jet is slower than that of the coherent jet, and the velocity core length of the main oxygen jet is extended by 30%. The SC coherent jet denotes a strong stirring intensity and impact capacity to the molten bath.

This study provides some basic data for the supersonic combustion coherent jet technology. However, further research is required to investigate supersonic combustion of the shrouding methane gas, including the flow rate, Mach number, and aperture of the supersonic shrouding gas, because these factors affect the supersonic combustion coherent jet characteristics; future research will focus on addressing this topic.

## Figures and Tables

**Table 1:** Boundary conditions.

Name of the Boundary	Type of Boundary Conditions	Values
Supersonic main oxygen inlet	mass flow rate	0.8 kg·s^−1^
	mass fractions	O_2_ = 100%
	total temperature	300 K
Supersonic shrouding gas inlet	Mach number	2/0.8
	mass fractions	CH_4_ = 100%
	temperature	300 K
Pressure inlet	static pressure	101,325 Pa
	mass fractions	O_2_ = 21%, N_2_ = 79%
	temperature	1700 K/300 K
Pressure outlet	static pressure	101,325 Pa
	mass fractions	O_2_ = 21%, N_2_ = 79%
	temperature	1700 K/300 K
Wall	no-slip	300 K

## References

[1] Eshwar K.R., Ville V.V., Petri S. (2019). Numerical modelling of the influence of argon flow rate and slag layer height on open-eye formation in a 150 ton steelmaking ladle. Metals.

[2] Zhang B., Chen K., Wang R.F. (2019). Physical modelling of splashing triggered by the gas jet of an oxygen lance in a converter. Metals.

[3] Odenthal H.J., Kemminger A., Krause F. (2017). Review on modeling and simulation of the electric arc furnace (EAF). Steel Res. Int..

[4] Ersson M., Tilliander A. (2018). Review on CFD simulation and modeling of decarburization processes. Steel Res. Int..

[5] Kärnä A., Järvinen M., Fabritius T. Supersonic lance mass transfer modelling. Proceedings of the International Conference on Physical and Numerical Simulation of Materials Processing.

[6] Anderson J.E., Farrenkopf D.R. (1998). Coherent Gas Jet. U.S. Patent.

[7] Ersson M., Tilliander A., Jonsson L. (2008). A mathematical model of an impinging air jet on a water surface. ISIJ Int..

[8] Sabah S., Brooks G. (2015). Splash distribution in oxygen steelmaking. Metall. Mater. Trans. B.

[9] Malfa E., Giavani C., Memoli F. (2005). Numerical simulation of a supersonic oxygen lance for industrial application in EAFs. MPT Int..

[10] Zhao F., Sun D., Zhu R. (2017). Effect of shrouding gas parameters on characteristics of supersonic coherent jet. Metall. Mater. Trans. B.

[11] Meidani A.R.N., Isac M., Richardson A. (2004). Modelling shrouded supersonic jets in metallurgical reactor vessels. ISIJ Int..

[12] Tang G.W., Chen Y., Silaen A.K. (2019). Effects of fuel input on coherent jet length at various ambient temperatures. App. Ther. Eng..

[13] Tang G.W., Chen Y., Silaen A.K. (2018). Investigation on coherent jet potential core length in an electric arc furnace. Steel Res. Int..

[14] Odenthal H.J., Bader J., Nörthemann R. The new generation of SIS injector for improved EAF processes. Proceedings of the METEC & 2nd ESTAD–European Steel Technology and Application Conference.

[15] Sumi I., Kishimoto Y., Kikuchi Y. (2006). Effect of high-temperature field on supersonic oxygen jet behavior. ISIJ Int..

[16] Sumi I., Okuyama G., Nabeshima S. (2007). Behavior of top-blown jet under reduced pressure. ISIJ Int..

[17] Klioutchnikov I., Olivier H., Odenthal J. (2013). Numerical investigation of coaxial jets entering into a hot environment. Comp. Fluids.

[18] Alam M., Naser J., Brooks G. (2010). Computational fluid dynamics modeling of supersonic coherent jets for electric arc furnace steelmaking process. Metall. Mater. Trans. B.

[19] Alam M., Naser J., Brooks G. (2010). Computational fluid dynamics simulation of supersonic oxygen jet behavior at steelmaking temperature. Metall. Mater. Trans. B.

[20] Alam M., Naser J., Brooks G. (2018). A computational fluid dynamics model of shrouded supersonic jet impingement on a water surface. ISIJ Int..

[21] Liu F., Sun D., Zhu R. (2017). Effect of shrouding gas temperature on characteristics of a supersonic jet flow field with a shrouding Laval nozzle structure. Metall. Mater. Trans. B.

[22] Liu F., Sun D., Zhu R. (2019). Effect of shrouding Mach number and ambient temperature on the flow field of coherent jet with shrouding Laval nozzle structure. Can. Metall. Q..

[23] Anderson J.D. (2013). Introduction to Flight.

[24] Versteeg H.K., Malalasekera W. (2007). An Introduction to Computational Fluid Dynamics, the Finite Volume Method.

[25] Abdolhamid K.S., Pao S.P., Massey S.J. (2006). Temperature corrected turbulence model for high temperature jet flow. J. Fluids Eng..

[26] Menter F.R. (1994). Two-equation eddy-viscosity turbulence models for engineering applications. AIAA J..

[27] Liu F., Zhu R., Dong K. (2016). Flow field characteristics of coherent jet with preheating oxygen under various ambient temperatures. ISIJ Int..

[28] Jones W.P., Whitelaw J.H. (1982). Calculation methods for reacting turbulent flows: A review. Combust. Flame.

[29] Papamoschou D., Roshko A. (1988). The compressible turbulent shear layer: An experimental study. J. Fluid Mech..

